# Outcomes of Late-Onset Bleb-Related Endophthalmitis Treated with Pars Plana Vitrectomy

**DOI:** 10.1155/2015/923857

**Published:** 2015-10-01

**Authors:** Kazuyoshi Ohtomo, Chihiro Mayama, Takashi Ueta, Miyuki Nagahara

**Affiliations:** Department of Ophthalmology, The University of Tokyo Hospital, 7-3-1 Hongo, Bunkyo-ku, Tokyo 113-8655, Japan

## Abstract

*Purpose*. To investigate clinical results on bleb-related endophthalmitis (BRE) after trabeculectomy treated with pars plana vitrectomy (PPV) and to evaluate influence factors for visual prognosis. *Methods*. Investigating medical records retrospectively, BRE was defined as an endophthalmitis induced by bleb infection. A total of 2018 eyes of 1225 patients who had trabeculectomy between December 2000 and July 2013 were included in this study. Eleven eyes of 11 patients with BRE were performed with PPV. *Results*. The mean age was 56.6 years. The mean period between trabeculectomy and BRE onset was 7.4 years. The mean period from starting symptom to initiation of treatment for endophthalmitis (PSITE) was 2.3 days. Bleb leakages were observed in 7 eyes (64%). On culture examinations, highly pathogenic bacteria (HPB) were identified in 6 cases (55%). HPB infection was influence factors on visual disturbance (*P* = .0337). Number of HPB infections is significantly higher in poor visual outcome than without poor visual outcome (*P* = .0310). *Conclusion*. Visual prognosis of BRE treated by PPV is significantly better when the pathogenic bacteria are not HPB. Severe visual loss occurred with HPB infection even though patients had appropriate treatments. Physicians need to have careful consideration to prevent bleb infection after trabeculectomy with MMC.

## 1. Introduction

Although glaucoma treatments have been developed, glaucoma is still a major cause of worldwide blindness. Trabeculectomy has long been performed as the only surgical procedure to achieve very low intraocular pressure (IOP) to prevent progression of visual field damage in glaucoma eyes [1].

However, there is a serious concern for bleb-related endophthalmitis (BRE) even after successful trabeculectomy, especially when antifibrotic agents including mitomycin C (MMC) are used during and after surgery. Effects of a nucleic acid metabolic inhibition by the antifibrotic agents are favorable for maintenance of functional bleb, whereas use of those agents is associated with formation of thin and avascular blebs which can be a risk factor of developing intraocular infection through the blebs [2–4]. This BRE can occur after early postoperative period, even ten years or more after the surgery, and annual incidence of BRE has been reported to range from 1.1 to 5.4% [3, 4]. Most of the eyes with BRE require urgent and intensive treatments including pars plana vitrectomy (PPV). It has been reported that visual prognosis was better in BRE cases treated with PPV rather than those treated with only medications and general, topical, and intravitreal administration of antibiotics [5–7]. To our knowledge, however, it has not been investigated which factors have a significant influence on the visual outcome in BRE eyes treated with PPV.

In the present study, we retrospectively reviewed clinical data of consecutive BRE cases after trabeculectomy with MMC which were treated with PPV and evaluated the relationship between visual prognosis and influence factors including species of pathogens and time interval between onset of BRE and therapeutic PPV.

## 2. Patients and Methods

This retrospective case series was approved by the ethics committee of The University of Tokyo Hospital and was conducted in accordance with the tenets of the Declaration of Helsinki.

**Figure 1:**
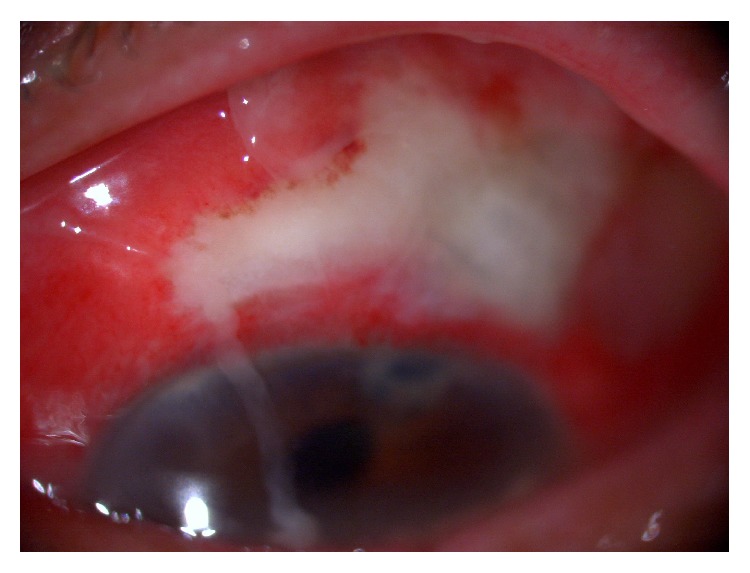
Anterior segment photography in patient with bleb-related endophthalmitis. BRE symptoms including hyperemia, pain, discharge, hypopyon, and loss of vision and the BRE diagnosis were identified as infectious inflammations spread to the vitreous body with white bleb.

According to the operation records and medical records, eyes that underwent trabeculectomy with intraoperative use of MMC between December 2000 and July 2013 and that were followed up at least for one year after BRE occurred at The University of Tokyo Hospital were included in this study. Eyes that had bleb infection without vitreous inflammation or vitreous inflammation without bleb infection and that underwent implantation of a glaucoma drainage device before occurrence of BRE within the periods were excluded from this study. The onset time of BRE was defined as a point when symptoms including hyperemia, pain, discharge, and loss of vision and the BRE diagnosis were identified as infectious inflammations spread to the vitreous body with white bleb infection [8] (Figure 1).

**Figure 2:**
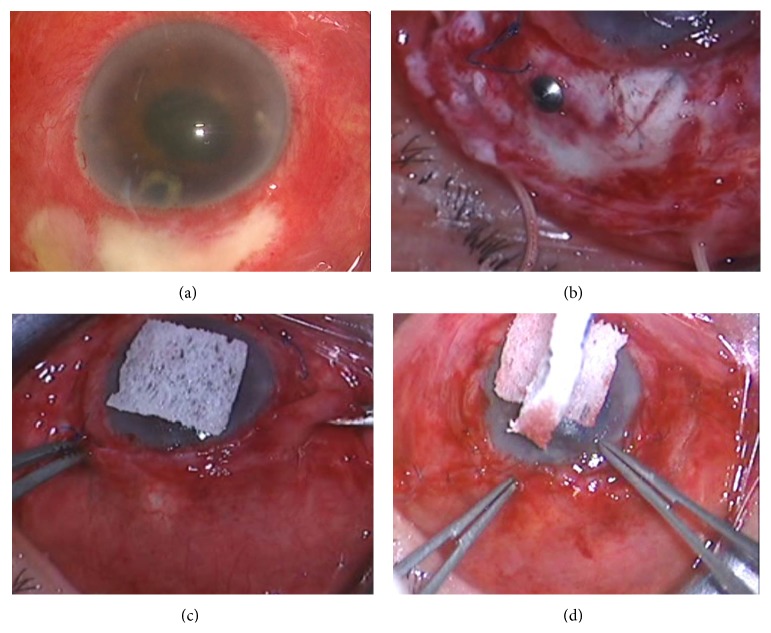
Surgical procedure of the infected bleb excision and conjunctival advancement. All figures are surgeon's view. (a) The period from starting symptom to initiation of treatment is 1 day. The white bleb opacity, the conjunctival hyperemia, and the cloudy anterior chamber with inflammation can be seen. (b) The infected bleb is removed completely after pars plana vitrectomy. (c) Conjunctival advancement is performed after sufficient posterior peritomy. The scleral flap was covered with the moved residual conjunctiva from the fornix to the limbus. (d) The moved conjunctiva was sutured at the limbus by a 10-0 nylon suture with a rounded-body needle.

As a general procedure of vitreous surgery, PPV was performed by using 20-gauge vitrector (ACCURUS, Alcon, Texas, USA) with making complete posterior vitreous detachment. If infectious inflammations spread to peripheral retina and severe vitreous base opacity were seen, scleral encircling (AU-240, AU-270; MIRA, Massachusetts, USA) and peripheral retinal photocoagulation were performed to avoid retinal detachment with occurrence of retinal tear induced by retinal necrosis after surgery. The infected bleb was excised for debridement at the end of surgery and conjunctival advancement was performed for covered scleral flap to avoid bleb-related complications [9]. Antibiotic agents were administrated during surgery with irrigating solution and also intravenous drip infusion and eye drop instillation were performed after surgery (Figure 2).

Surgical and medical treatment such as lensectomy, retinal laser treatment, scleral encircling, conjunctival advancement, the usage of antibiotics during PPV, and the systemic, local administration after PPV were dependent on the eye and general conditions. In this study, the periods from starting symptom of BRE to initiation of PPV for endophthalmitis were defined as the periods from starting symptom to initiation of treatment for endophthalmitis (PSITE) to evaluate association between times and visual prognosis. The visual acuity, IOP, and posttreatment complications were obtained by patient records. Visual acuities after PPV were evaluated with the final visual acuity. The IOP was evaluated with the last IOP before BRE and the final IOP after PPV.

As for pathogens identified in culture examination, we classified them into highly pathogenic bacteria (HPB) or others. In the present study, HPB were constituted of* Streptococcus* species,* Enterococcus faecalis*, and Gram-negative bacteria including* Pseudomonas aeruginosa* and* Haemophilus influenza* that produce highly toxic exotoxins and reportedly associated with poor prognosis for endophthalmitis [10–12]. For culture of pathogens, intraocular lens (IOL), undiluted aqueous humor, and undiluted vitreous samples were always obtained under sterile condition during surgery.

All culture results were obtained using the standard microbiologic procedures using thioglycolate liquid, chocolate agar (37°C, in CO_2_), and enriched blood agar (37°C, in CO_2_) as culture media. Standard biochemical testing methods were used for speciation of cultured bacteria.

## 3. Statistics

The values of decimal BCVA scores were converted into logarithm of the minimum angle of resolution (logMAR) values. Visual acuities of counting finger (CF), hand motion (HM), light perception (LP), and no light perception (NLP) were assigned to logMAR values of 2, 3, 4, and 5, respectively. Statistical analysis was performed using JMP9 software (SAS Inc., Cary, NC). Wilcoxon's signed-rank test and Wilcoxon's rank sum test were used to analyze numerical data. Fisher's exact test was used to analyze categorical data. A *P* value of <.05 was regarded as statistically significant.

## 4. Results

A total of 2018 eyes of 1225 patients that underwent trabeculectomy with intraoperative MMC between December 2000 and July 2013 were included in this study. Forty eyes had bleb-related infection and 14 eyes were diagnosed with BRE within the periods. Three eyes were excluded because they had no whitened bleb infection. Eleven eyes undergoing PPV were chosen as development of BRE.

Characteristics of the 11 subjects of this study are detailed in Table 1. The age at BRE onset was from 30 to 81 years. Five (45%) patients were male and 6 patients (55%) were female. The follow-up period after BRE was from 2 months to 8.6 years, with a mean of 3.3 years. The period between trabeculectomy and BRE onset was from 1 to 16 years, with a mean of 7.4 years. The PSITE was from 0 to 7 days, with a mean of 2.3 days.

Bleb leakages were observed in 4 eyes (36%) with the Seidel test when BRE occurred. The bleb was located in inferior half of the limbus in only 1 eye (9%). There were 2 patients with connective tissue disease including each one case of Sjögren's syndrome and dermatomyositis and 1 patient with diabetes mellitus. Oral corticosteroid was prescribed for 1 patient with dermatomyositis. The lens condition was phakia, pseudophakia, or aphakia in 5 eyes (45%), 5 eyes (45%), and 1 eye, respectively.

Four (36%) of the 11 patients received PPV with IOL removal. Three patients (27%) received PPV with lensectomy. Six (55%) patients underwent intraoperative laser treatment. There was no patient treated with scleral encircling simultaneously to prevent retinal detachment.

There were significantly no differences on the IOP in 11 patients performed with bleb removal and conjunctival advancement before and after surgery. The number of antiglaucoma medications was from 0 to 3. Seven patients (64%) had no antiglaucoma medication and well controlled IOP. Four patients (36%) required antiglaucoma medications; however, 2 (18%) of them were uncontrollably high IOP (>21 mmHg) with antiglaucoma medications after BRE. One patient with uncontrolled IOP wished to observe without performing glaucoma surgery. The other patient continued high IOP instead of performing with trabeculectomy after 2 months from BRE.

Intravitreously administrated antibiotics during surgery were vancomycin and ceftazidime (82%), ceftazidime alone (9%), and imipenem alone (9%). Antibiotic eye drops after PPV were also used in all patients and administered in combination including levofloxacin (64%), gatifloxacin (21%), cefmenoxime (82%), sulpelin (9%), erythromycin (9%), tobramycin (9%), and vancomycin (18%). Systemic antibiotics after PPV were used in 5 patients including imipenem alone (27%), flomoxef alone (9%), and imipenem and ceftazidime (9%).

Repeated PPV was needed at 9 days after the initial PPV in one eye because of persistent intravitreous inflammation while BRE was controlled after the initial PPV in the other patients. In contrast, another treatment was needed in 2 eyes. One eye was performed with phacoemulsification because of phacolytic endophthalmitis after 3 months from BRE and the other eye was performed with PPV because of proliferative vitreoretinopathy after a month from BRE. Those conditions were not infection related. Bullous keratopathy was developed in 4 eyes (36%) and another one eye finally resulted in phthisis bulbi.

In the present case, series organisms were identified from 8 patients (73%), while in 3 patients no organism was detected in any samples. All organisms isolated were Gram-positive bacteria.* Streptococcus* species were identified in samples of 5 patients including 2 patients with* Streptococcus pneumoniae*, 2 with* Streptococcus mitis*, and 1 with unidentifiable* Streptococcus*.* Enterococcus faecalis* was isolated from 1 patient.* Staphylococcus epidermidis* was isolated from 1 patient. Unidentifiable Gram-positive bacteria were isolated from 1 patient. In total, HPB were identified in samples of 6 patients (55%). There is no detection on resistive bacteria in these cases.

The incubation of all specimens' results is shown in Table 2.

All specimens of 11 patients were 26 samples. In all of 11 cases, conjunctival incubation was performed. Incubation of aqueous humor in 5 eyes, removed IOL in 2 eyes, and vitreous humor in 8 eyes was performed. In conjunctiva, the bacteria were detected in 4 (36%) of 11 specimens. The HPB were 2 specimens including* Streptococcus pneumoniae* and group A* Streptococcus*. But the detected bacteria without HPB were unidentifiable Gram-positive bacteria in 2 specimens. In aqueous humor, the bacteria were detected in 3 (60%) of 5 specimens. The HPB were 2 specimens including* Streptococcus pneumoniae* and group A* Streptococcus*. In contrast, the detected bacteria without HPB were* Staphylococcus epidermidis* in 1 specimen. In removed IOL, the bacteria were detected in 1 (50%) of 2 specimens including unidentifiable* Streptococcus* as HPB. In vitreous humor, the bacteria were detected in 3 (38%) of 8 specimens. The HPB were 2 specimens including* Streptococcus pneumoniae* and* Enterococcus faecalis*. However, the detected bacteria without HPB were unidentifiable Gram-positive bacteria in 1 specimen.

From HPB infection's point of view, these cases were classified into 2 groups with HPB infection and without HPB infection (Table 3); HPB group was significantly higher than those without HPB on the parameters of age (*P* = .0349) and the last BCVA after PPV for BRE (*P* = .0337).

Even though PSITE was within 1 day, severe visual loss occurred in* Enterococcus faecalis* infection. The other parameters including gender, bleb leakage, bleb location, lens condition, diabetes mellitus, connective tissue disease, oral corticosteroids, and ophthalmic past history were not significantly associated.

Finally, these cases were classified into 2 groups: with poor visual outcome (logMAR values ≥2) and without poor visual outcome (logMAR values <2).  Table 4 shows total number of developments of BRE and number of BREs with poor visual outcome. The poor visual outcome group was significantly higher than the without poor visual outcome group on the parameter of HPB infection (*P* = .0310).

The other parameters including gender, age, diabetes mellitus, connective tissue disease, oral administration of corticosteroids, lens status, blepharitis, reoperation trabeculectomy before BRE, bleb location, use of antimetabolites, type of conjunctival flap, conjunctival suture with silk, postoperative complications (early leak, choroidal effusion, hypotony, late onset leak, and cataract surgery before BRE), postoperative bleb manipulation, and PSITE were not significantly different.

## 5. Discussion

According to a recent multicenter prospective study in Japan, accumulative incidence of bleb-related infection, which included BRE and more early phase of the infection of which inflammation was limited within bleb or anterior chamber, was 2.2% in 5-year follow-up period after trabeculectomy. PPV was required in 14.3% of those bleb-related infection cases for treatment [2]. The purpose of this study was to investigate outcome of PPV treatment to BRE. PPV was selected on treatment of BRE in severe infection. There is a clinically significant if we can predict patient's visual prognosis as the predisposing factors such as timing of PPV or the other factors. Multicenter prospective study cannot estimate those factors and also previous studies have not been investigated.

In the present study, the mean age at the time of presentation with endophthalmitis was 56.6 years, with a range of 30 to 81 years. Several reports have indicated age under 60 years as a risk factor for the bleb-related infection [2, 13]. This was speculated because younger patients were more physically active, which made their blebs in higher risk for the exposure to infective agents or trauma.

In previous studies, the mean period from initial trabeculectomy to bleb-related infection was from 3.1 to 5 years [2, 3, 6]. In our study, the interval from trabeculectomy to the onset of BRE ranged from 1 to 16 years, with a mean of 7.4 years. The results suggest that careful observation may be required indefinitely after trabeculectomy. In our study, the bleb leakages were noted in 64% when BRE occurred. The bleb leakages have been reported as a risk factor of the bleb-related infection [2, 14]. Inferior bleb location was noted in 9%. Inferior bleb has also been considered as a risk factor of the bleb-related infection because of being uncovered with the eyelid and infected by bacteria accumulated in conjunctival sac [15, 16].

As the visual outcome after treatment with PPV, previous studies showed the median log MAR value of the last BCVA was from 1 to 2 [5, 7]. In the present study, median log MAR value of the final BCVA was showed as 0.82.

Although BRE is an emergency and should be managed aggressively, there is no evidence about how early BRE should be treated by PPV. In our findings, the last BCVA after BRE was significantly better when the responsible pathogens are not HPB. BRE causes severe vision loss in HPB infection even in patients who received PPV within 24 hours.

As for the bleb removal and conjunctival advancement well known to be useful for stopping the bleb leakage [9, 17], there was no detail report to apply in eyes with BRE to our knowledge. In our study, we required the bleb removal and conjunctival advancement because of the bleb with white opacity which is suspected with infectious cause. Because the bleb leakage is risk factor for BRE and the bleb infection recurs with high frequency in the past history of bleb infection [2, 14, 18], we recommend this procedure for the patient who needs treatment with PPV for BRE.

As for the prognosis of IOP after PPV, there were few previous studies to mention the prognosis of IOP after BRE including treatment with and without PPV because of the miserable ocular statements such as enucleation, phthisis bulbi, and bullous keratopathy [5–7, 19–22]. In previous studies, the mean IOP was 13-14 mmHg and the IOP remained controlled without medication or with no increase in medication in 53-54% [6, 19, 20]. Eventually, the IOP was uncontrolled after treatment of endophthalmitis in 11% of the cases in spite of medications or glaucoma shunt surgery [6]. Our results of those were similar to previous studies.

The most common bacteria that cause BRE include Streptococcal species,* Haemophilus influenza*, and* Staphylococcus* species [7, 15]. As for the correlations between the cultured bacteria and BCVA after BRE, the visual outcome is poor when BREs occur, especially with virulent bacteria such as Streptococcal species [5],* Enterococcus faecalis* [11], coagulase-positive* Staphylococci*, or Gram-negative bacteria. In contrast, coagulase-negative Staphylococcal bacteria such as* Staphylococcus epidermidis* tend to have a more benign course [15]. The results of bacterial culture in the present study were similar to those of these previous reports. In our study, we evaluated influence of pathogenic bacteria on visual disturbance (Table 3), and it showed that the types of bacteria may be associated with the visual prognosis. Furthermore, these cases were classified into 2 groups: with poor visual outcome or no visual outcome and without poor visual outcome (Table 4), and it showed that HPB may be associated with the visual prognosis.

Limitations of the present study include that the present study was retrospective, evaluated a small number of patients, and was conducted in a single hospital, although we searched the hospital record of 13 years.

In conclusion, the present study suggests that visual prognosis of BRE treated by PPV is significantly better when the pathogenic bacteria are not HPB. Severe visual loss occurred with HPB infection even though patients had appropriate treatments for bleb-related complications. Physicians need to have careful consideration to prevent bleb infection after trabeculectomy with MMC.

## Figures and Tables

**(a):** 

Case	Gender	Age (years)	Type of glaucoma	PSITE (days)	HPB (+/−)	Bacteria/location	BCVA before infection (log⁡MAR value)	BCVA after surgery (log⁡MAR value)	IOP before BRE (mmHg)	IOP after surgery (mmHg)
1	Male	62	SOAG (Uveitis)	0	+	*Enterococcus faecalis*/V	0.10	3	8	12
2	Female	56	SOAG	2	+	*S. pneumoniae*/C	0.70	5	5	0
3	Male	73	POAG	2	+	*S. pneumoniae*/V	0.52	5	6	7
4	Male	53	SOAG (Uveitis)	2	+	Group A *Str*/C, A	−0.08	1.70	6	18
5	Female	68	SOAG (steroid)	3	+	Unidentifiable *Str*/I GPR/C, V	0.40	0.40	14	16
6	Female	81	Congenital	7	+	*S. mitis*/A	1.52	5	7	2
7	Female	40	Congenital	0	−	No growth	0.22	0.70	15	30
8	Male	41	POAG	0	−	*S. epidermidis*/A	0	0	10	11
9	Female	62	SOAG	1	−	No growth	0.52	0.70	9	11
10	Female	30	Congenital	3	−	No growth	0.15	0.82	11	22
11	Male	53	POAG	4	−	GPR/C	−0.08	0.30	10	10

**(b):** 

Case	Follow-up periods (years)	Period from LET to BRE (years)	General complications	Bleb leakage	Intraoperative laser treatment	Intravitreously administrated antibiotics during surgery	Antibiotic eye drops after surgery	Systemic antibiotics after PPV	Phakia, IOL, and aphakia
1	4.9	11	—	−	+	Vancomycin/ceftazidime	Levofloxacin/cefmenoxime	—	Phakia
2	5.0	2	Sjögren's syndrome	+	+	Vancomycin/ceftazidime	Levofloxacin/cefmenoxime	—	Phakia
3	5.8	5	—	−	+	Vancomycin/ceftazidime	Levofloxacin/cefmenoxime	—	IOL
4	8.6	3	—	+	+	Imipenem	Sulpelin/erythromycin/ tobramycin	—	IOL
5	3.8	6	Dermatomyositis	−	+	Vancomycin/ceftazidime	Levofloxacin	Imipenem	IOL
6	0.2	16	Axenfeld-Rieger syndrome	+	−	Ceftazidime	Levofloxacin/cefmenoxime/ tobramycin/vancomycin	Imipenem/ ceftazidime	Phakia
7	0.7	7	—	−	−	Vancomycin/ceftazidime	Levofloxacin/cefmenoxime	Imipenem	Phakia
8	1.3	6	—	+	−	Vancomycin/ceftazidime	Gatifloxacin/cefmenoxime	—	IOL
9	3.0	1	Diabetic mellitus	+	−	Vancomycin/ceftazidime	Gatifloxacin/cefmenoxime/ vancomycin	Imipenem	IOL
10	2.6	17	—	+	−	Vancomycin/ceftazidime	Gatifloxacin/cefmenoxime	Flomoxef	Aphakia
11	0.8	9	—	+	+	Vancomycin/ceftazidime	Levofloxacin/cefmenoxime	—	Phakia

SOAG = secondary open angle glaucoma, POAG = primary open angle glaucoma, PSITE = the periods from starting symptom to initiation of treatment for endophthalmitis, HPB = highly pathogenic bacteria, V = vitreous specimen, C = conjunctival specimen, A = anterior chamber's specimen, I = specimen of removed intraocular lens, BCVA = best corrected visual acuity, IOP = intraocular pressure, BRE = bleb-related endophthalmitis, *S. pneumoniae* = *Streptococcus pneumoniae*, group A *Str* = group A *Streptococcus*, *S. mitis* = *Streptococcus mitis*, unidentifiable *Str* = unidentifiable *Streptococcus*, group G *Str* = group G *Streptococcus*, *S. epidermidis* = *Staphylococcus epidermidis*, LET = trabeculectomy, PPV = pars plana vitrectomy, and IOL = intraocular lens.

**Table 2:** All specimens and distribution and details of specimens.

Location	Number of specimens	Number of detected specimens	HPB	Without HPB
Conjunctiva	11	4	*S. pneumoniae *Group A *Str*	Unidentifiable GPB (2)

Aqueous humor	5	3	*S. mitis *Group A *Str*	*S. epidermidis*

IOL	2	1	Unidentifiable *Str*	

Vitreous humor	8	3	*S. pneumoniae E. faecalis*	Unidentifiable GPB

HPB = highly pathogenic bacteria, *S. pneumoniae* = *Streptococcus pneumoniae*, group A *Str* = group A *Streptococcus*, *S. mitis* = *Streptococcus mitis*, unidentifiable *Str* = unidentifiable *Streptococcus*, *E. faecalis* = *Enterococcus faecalis*, unidentifiable GPB = unidentifiable Gram-positive bacteria, and *S. epidermidis* = *Staphylococcus epidermidis*.

**Table 3:** Comparison with or without highly pathogenic bacteria.

	HPB (6)	Without HPB (5)	*P* value
Age (year, mean ± SD)	65.8 ± 10.7	45.5 ± 12.5	.0349^*∗*^
Male/female	3/3	2/3	1.0000
Right/left	1/5	1/4	1.0000
Median log⁡MAR value before BRE	0.46	0.15	.3131
Median log⁡MAR value after PPV	4.0	0.7	.0337^*∗*^
IOP before BRE (mmHg, mean ± SD)	7.7 ± 3.3	11.0 ± 2.3	.0541
IOP after PPV (mmHg, mean ± SD)	9.2 ± 7.4	16.8 ± 8.9	.3591
Follow-up period after BRE (year, mean ± SD)	4.7 ± 2.8	1.7 ± 1.1	.0828
Period between trabeculectomy and BRE onset (year, mean ± SD)	7.2 ± 5.3	7.8 ± 5.5	.7832
PSITE (day, mean ± SD)	2.7 ± 2.3	1.5 ± 1.7	.6427
Bleb leakage (when BRE occurred)	3	4	.5455
Bleb location (superior/inferior)	5/1	5/0	1.0000
Lens condition (with phakia/without phakia)	3/3	2/3	1.0000
Diabetes mellitus	1	1	1.0000
Connective tissue disease	2	0	.4545
Oral administration of corticosteroids	1	0	1.0000
Previous trabeculectomy (time, mean ± SD)	1.5 ± 0.5	1.4 ± 0.9	.5925
Previous intraocular surgery (time, mean ± SD)	1.8 ± 1.0	2.6 ± 1.1	.2911
Bleb removal and conjunctival advancement	6	5	1.0000
PPV with LASER treatment	5	1	.0801

^*∗*^Wilcoxon's signed-rank test.

Without phakia means pseudophakia or aphakia.

HPB = highly pathogenic bacteria, SD = standard deviation, BRE = bleb-related endophthalmitis, PPV = pars plana vitrectomy, IOP = intraocular pressure, and PSITE = the periods from starting symptom to initiation of treatment for endophthalmitis.

**Table 4:** Comparison factors between BREs with and without poor visual outcome.

Parameter	Number of BREs	Number of BREs with poor visual outcome (%)	*P* value
Demographic characteristics			
Sex			
Male	8	3 (38)	1.0000
Female	6	2 (33)	
Age (old age ≤ 60)			
Young age	7	1 (14)	0.2657
Old age	7	4 (57)	
Systemic conditions			
Diabetes mellitus			
Yes	2	0	0.5055
No	12	5 (42)	
Connective tissue disease			
Yes	3	1 (33)	1.0000
No	11	4 (36)	
Oral administration of corticosteroids			
Yes	2	0	0.5055
No	12	5 (42)	
Lens status			
Phakia	6	3 (67)	0.0909
IOL or aphakia	8	2 (13)	
Blepharitis			
Yes	2	1 (50)	1.0000
No	12	4 (33)	
Surgical factors			
Reoperation trabeculectomy before BRE			
Yes	4	2 (50)	0.5804
No	10	3 (30)	
Bleb location			
Superior	13	4 (31)	0.3571
Inferior	1	1 (100)	
Antimetabolites (MMC)			
Yes	14	5 (36)	NA
No	0	0	
Type of conjunctival flap			
Fornix base	2	1 (50)	1.0000
Limbus base	12	4 (33)	
Conjunctival suture with silk			
Yes	0	0	NA
No	14	5 (36)	
Postoperative complications			
Early leak			
Yes	0	0	NA
No	14	5 (36)	
Choroidal effusion			
Yes	0	0	NA
No	14	5 (36)	
Hypotony (<8 mmHg)			
Yes	4	3 (75)	0.0949
No	10	2 (20)	
Late on-set leak			
Yes	6	2 (33)	1.0000
No	8	3 (38)	
Cataract surgery before BRE			
Yes	4	1 (25)	1.0000
No	10	4 (40)	
Postoperative bleb manipulation (revision or needling)			
Yes	6	2 (33)	1.0000
No	8	3 (38)	
PSITE			
≥1 day	12	4 (33)	1.0000
<1 day	2	1 (50)	
Highly pathogenic bacteria infection			
Yes	8	5 (63)	0.0310^*∗*^
No	6	0	

NA = not applicable.

^*∗*^Fisher's exact test.

## References

[1] Musch D. C., Gillespie B. W., Lichter P. R., Niziol L. M., Janz N. K. (2009). Visual field progression in the Collaborative Initial Glaucoma Treatment Study. The impact of treatment and other baseline factors. *Ophthalmology*.

[2] Yamamoto T., Sawada A., Mayama C. (2014). The 5-year incidence of bleb-related infection and its risk factors after filtering surgeries with adjunctive mitomycin C: collaborative bleb-related infection incidence and treatment study 2. *Ophthalmology*.

[3] Mochizuki K., Jikihara S., Ando Y., Hori N., Yamamoto T., Kitazawa Y. (1997). Incidence of delayed onset infection after trabeculectomy with adjunctive mitomycin C or 5-fluorouracil treatment. *British Journal of Ophthalmology*.

[4] DeBry P. W., Perkins T. W., Heatley G., Kaufman P., Brumback L. C. (2002). Incidence of late-onset bleb-related complications following trabeculectomy with mitomycin. *Archives of Ophthalmology*.

[5] Busbee B. G., Recchia F. M., Kaiser R. (2004). Bleb-associated endophthalmitis: clinical characteristics and visual outcomes. *Ophthalmology*.

[6] Song A., Scott I. U., Flynn M. P. H. H. W., Budenz D. L. (2002). Delayed-onset bleb-associated endophthalmitis: clinical features and visual acuity outcomes. *Ophthalmology*.

[7] Kangas T. A., Greenfield D. S., Flynn H. W., Parrish R. K., Palmberg P. (1997). Delayed-onset endophthalmitis associated with conjunctival filtering blebs. *Ophthalmology*.

[8] Yamamoto T., Kuwayama Y., Kano K., Sawada A., Shoji N. (2013). Clinical features of bleb-related infection: a 5-year survey in Japan. *Acta Ophthalmologica*.

[9] Budenz D. L., Chen P. P., Weaver Y. K. (1999). Conjunctival advancement for late-onset filtering bleb leaks: indications and outcomes. *Archives of Ophthalmology*.

[10] Gupta A., Orlans H. O., Hornby S. J., Bowler I. C. J. W. (2014). Microbiology and visual outcomes of culture-positive bacterial endophthalmitis in Oxford, UK. *Graefe's Archive for Clinical and Experimental Ophthalmology*.

[11] Scott I. U., Loo R. H., Flynn H. W., Miller D. (2003). Endophthalmitis caused by *Enterococcus faecalis*: antibiotic selection and treatment outcomes. *Ophthalmology*.

[12] Eifrig C. W. G., Scott I. U., Flynn H. W., Miller D. (2003). Endophthalmitis caused by *Pseudomonas aeruginosa*. *Ophthalmology*.

[13] Wolner B., Liebmann J. M., Sassani J. W., Ritch R., Speaker M., Marmor M. (1991). Late bleb-related endophthalmitis after trabeculectomy with adjunctive 5-fluorouracil. *Ophthalmology*.

[14] Matsuo H., Tomidokoro A., Suzuki Y., Shirato S., Araie M. (2002). Late-onset transconjunctival oozing and point leak of aqueous humor from filtering bleb after trabeculectomy. *American Journal of Ophthalmology*.

[15] Azuara-Blanco A., Katz L. J. (1998). Dysfunctional filtering blebs. *Survey of Ophthalmology*.

[16] Soltau J. B., Rothman R. F., Budenz D. L. (2000). Risk factors for glaucoma filtering bleb infections. *Archives of Ophthalmology*.

[17] Burnstein A. L., WuDunn D., Knotts S. L., Catoira Y., Cantor L. B. (2002). Conjunctival advancement versus nonincisional treatment for late-onset glaucoma filtering bleb leaks. *Ophthalmology*.

[18] Lehmann O. J., Bunce C., Matheson M. M. (2000). Risk factors for development of post-trabeculectomy endophthalmitis. *British Journal of Ophthalmology*.

[19] Greenfield D. S., Suñer I. J., Miller M. P., Kangas T. A., Palmberg P. F., Flynn H. W. (1996). Endophthalmitis after filtering surgery with mitomycin. *Archives of Ophthalmology*.

[20] Waheed S., Ritterband D. C., Greenfield D. S., Liebmann J. M., Seedor J. A., Ritch R. (1998). New patterns of infecting organisms in late bleb-related endophthalmitis: a ten year review. *Eye*.

[21] Ciulla T. A., Beck A. D., Topping T. M., Baker A. S. (1997). Blebitis, early endophthalmitis, and late endophthalmitis after glaucoma-filtering surgery. *Ophthalmology*.

[22] Mandelbaum S., Forster R. K., Gelender H., Culbertson W. (1985). Late onset endophthalmitis associated with filtering blebs. *Ophthalmology*.

